# Clinical Effects of Oral Bacteriotherapy on Anal HPV Infection and Related Dysplasia in HIV-Positive MSM: Results from the “HPVinHIV” Trial

**DOI:** 10.3390/biomedicines9111738

**Published:** 2021-11-22

**Authors:** Eugenio Nelson Cavallari, Giancarlo Ceccarelli, Letizia Santinelli, Giuseppe Pietro Innocenti, Gabriella De Girolamo, Cristian Borrazzo, Ornella Spagnolello, Carolina Scagnolari, Stefano Arcieri, Antonio Ciardi, Alessandra Pierangeli, Claudio Maria Mastroianni, Gabriella d’Ettorre

**Affiliations:** 1 Department of Public Health and Infectious Diseases, “Sapienza” University of Rome, 00161 Rome, Italy; eugenionelson.cavallari@uniroma1.it (E.N.C.); letizia.santinelli@uniroma1.it (L.S.); giuseppepietro.innocenti@uniroma1.it (G.P.I.); gabriella.degirolamo@uniroma1.it (G.D.G.); cristian.borrazzo@uniroma1.it (C.B.); spagnolello.ornella@gmail.com (O.S.); claudio.mastroianni@uniroma1.it (C.M.M.); gabriella.dettorre@uniroma1.it (G.d.); 2 Azienda Ospedaliero-Universitaria Policlinico Umberto I, 00161 Rome, Italy; 3 Department of Molecular Medicine, “Sapienza” University of Rome, 00161 Rome, Italy; carolina.scagnolari@uniroma1.it (C.S.); alessandra.pierangeli@uniroma1.it (A.P.); 4 Department of Surgical Sciences, “Sapienza” University of Rome, 00161 Rome, Italy; stefano.arcieri@uniroma1.it; 5 Department of Radiology, Oncology and Human Pathology, “Sapienza” University of Rome, 00161 Rome, Italy; antonio.ciardi@uniroma1.it

**Keywords:** HPV, HIV, MSM, anal dysplasia, probiotics, oral bacteriotherapy

## Abstract

Background. Anal HPV infection, anal dysplasia and, ultimately, anal cancer are particularly common in HIV-infected men who have sex with men. Treatment of anal dysplasia, aiming to prevent evolution to squamous cell carcinoma of the anus, is currently limited to direct ablation and/or application of topical therapy. The aim of the present study is to investigate the effect of oral bacteriotherapy (Vivomixx® in EU, Visbiome® in USA) on anal HPV infection and HPV-related dysplasia of the anal canal in HIV-infected men who have sex with men. Methods. In this randomized, placebo-controlled, quadruple-blinded trial (NCT04099433), HIV-positive men who have sex with men with anal HPV infection and HPV-related dysplasia were randomized to receive oral bacteriotherapy or placebo for 6 months. Anal HPV test, anal cytology and high resolution anoscopy with biopsies of anal lesions were performed at baseline and at the end of the study. Safety and tolerability of oral bacteriotherapy were also evaluated. Interim analysis results were presented. Results. 20 participants concluded the study procedures to date. No serious adverse events were reported. In respect to participants randomized to placebo, individuals in the experimental arm showed higher rate of anal dysplasia regression (*p* = 0.002), lower rate of onset of new anal dysplasia (*p* = 0.023) and lower rates of worsening of persistent lesions (*p* = 0.004). Clearance of anal HPV infection was more frequently observed in the bacteriotherapy group (*p* = 0.067). Conclusion. Being an interim analysis, we limit ourselves to report the preliminary results of the current study. We refer the conclusions relating to the possible effectiveness of the intervention to the analysis of the definitive data.

## 1. Introduction

Squamous cell carcinoma of the anus (SCCA) is almost invariably associated with Human Papilloma Virus (HPV) infection in the anal canal and, similarly to cervical cancer, it is typically preceded by the onset of local dysplasia, namely: low grade and high grade squamous intraepithelial lesions (LSIL and HSIL, respectively) [1,2].

Although HPV infection, as well as LSIL and HSIL, can regress spontaneously, HIV infected individuals show high rates of HPV persistence and high incidence of dysplasia [3,4,5,6]. Not surprisingly, the incidence of anal SIL and SCCA is particularly high among HIV-infected men having sex with men (MSM), with receptive anal intercourse being a major risk factor for acquisition of anal HPV infection [7,8].

Modulation of microbial flora with the use of probiotics, either through oral or local administration, promoted the clearance of genital HPV infection and dysplasia among the HIV-negative female population in prospective not-controlled clinical trials [9,10,11,12]. The main mechanisms through which this effect is exerted are represented by the restoration of local microbiome and epithelial lining, immunomodulation of the local immune response and interference with pathogen colonization [13,14,15,16,17,18,19]. Notably, HIV-infected MSM almost invariably show alterations of the normal composition of the gut microbiome (dysbiosis) [20], which may lead to impaired local immune system and, therefore, could contribute to HPV persistence in the anal tract [21,22,23]. To the best of our knowledge, no study addressed the role of oral bacteriotherapy on the progression of HPV-related lesions of the anal canal so far. Our literature search retrieved only one case report describing the clearance of multiple anal condylomas in a 56-year-old HIV-infected man following local and oral bacteriotherapy [24]. We hypothesized that similar results might be achieved in the HIV+ MSM population through oral administration of high dose of probiotics.

Herein we report the interim analysis results of a randomized controlled trial (RCT) investigating the effect of oral bacteriotherapy on anal HPV infection and HPV-related dysplasia in highly active anti-retroviral therapy (HAART)-treated HIV+ MSM.

## 2. Materials and Methods

### 2.1. Study Design and Outcomes

**Figure 1:**
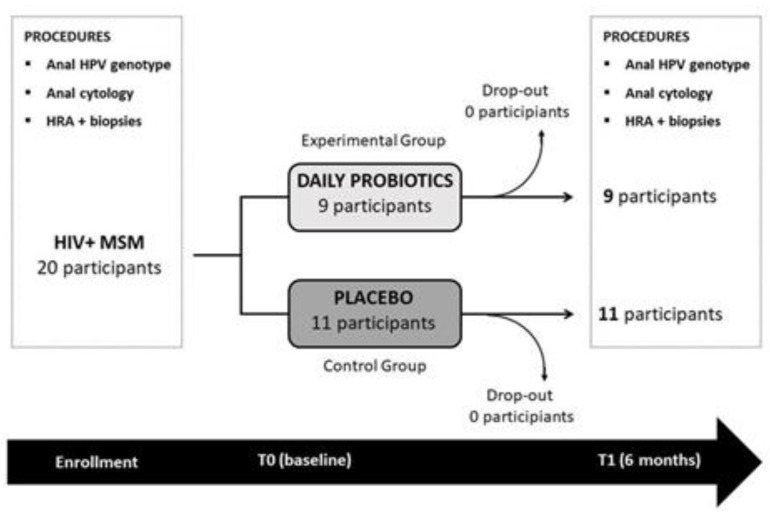
Study flow-chart.

The present randomized, placebo controlled, quadruple blind study (participants, care providers, investigators, outcome assessors), was designed to investigate the effect of oral bacteriotherapy on anal HPV infection and HPV-related dysplasia in HAART treated HIV+ MSM. Two study arms were compared: an arm formerly called “experimental group” in which oral bacteriotherapy was administered, and an arm called “control group” whose participants took a placebo. Randomization was independently carried out on a 1:1 basis by an external society. Study design is reported in Figure 1.

Primary outcomes were: (i) change from baseline in the number of HPV positive anal swabs, (ii) change from baseline in the number of dysplastic lesions.

The rate of adverse events throughout the study was evaluated as secondary outcome.

### 2.2. Eligibility and Enrollment Criteria

Participants were enrolled at the HIV Outpatient Clinic of Policlinico Umberto I Hospital of “Sapienza” University of Rome, Italy.

Subjects were considered eligible for the study if: (i) >18 years old; (ii) on stable HAART since at least 12 months with undetectable HIV RNA in plasma (lower limit of detection 37 copies/mL); (iii) positive for anal HPV infection with at least 1 histology proven area of anal dysplasia of any grade.

Exclusion criteria were: (i) refusal to provide written informed consent; (ii) known diagnosis of inflammatory bowel disease; (iii) use of antibiotics and/or probiotics within the 3 months prior to screening for enrollment in the study. To avoid the risk of bias, HPV vaccinated subjects were not enrolled and initiation of vaccination course during the study was not allowed. Similarly, the use of over-the-counter probiotics represented a reason for withdrawal from the study.

### 2.3. Study Procedures

Demographic and clinical data were collected at baseline (T0) and after 6 months (T1) on electronic CRF. At each study visit (T0 and T1) participants underwent: (1) anal HPV DNA detection and genotyping; (2) anal cytology; (3) high resolution anoscopy (HRA) with biopsies of areas suspicious for dysplasia. A self-reported questionnaire investigating the presence of gastrointestinal symptoms (abdominal bloating, abdominal pain, fecal consistency, number of daily evacuations, nausea, constipation) was administered to all participants at T0 and T1.

HPV DNA extraction was performed using QIAamp Blood and Tissue kit (Qiagen, Milano, Italy). HPV identification has been performed through PCR using a pair of specific primers: MY09 and MY11, which allowed the amplification of a 450 bp fragment of the late L1 region of HPV. PCR products corresponding to proper fragments were purified with QIAquick PCR purification kit. DNA sequencing was performed with an automatic DNA sequencer (model 370A Applied Biosystems, Monza, Italy), according to the manufacturer specifications (Amplicycle Kit, Applied Biosystems, Monza, Italy). Sequence similarity was determined by BLAST and ClustalW programs. Samples were classified as “multiple infection” when L1 and E6/7 sequencing gave discordant results [25].

Clearance of anal HPV infection was defined as: (a) negative swab at the end of the study in participants with positive swab at baseline, (b) positive swab at the end of the study that shows a different genotype from baseline [26].

Cytology specimens were collected over a period of 15–20 s, smeared onto a glass slide and fixed with alcohol within 15 s from brush withdrawal from the anus. The slides were stained using Papanicolau stain; cytology results were provided following the LAST recommendations [27].

HRA were conducted at T0 and T1 by the same provider. HRA technique and areas of suspected dysplasia were defined following the 2016 IANS International Guidelines for Practice Standards in the Detection of Anal Cancer Precursors [28]. Biopsies were performed on every area showing characteristics of possible dysplasia with the use of sterile disposable colonoscopy forceps. Biopsies were immediately fixed with formalin solution and sent to the Pathology Laboratory; histology results were provided following the LAST recommendations.

T1 HRA was planned within 30 days from the end of intake of the study product but due to the SARS-CoV2 pandemic it was delayed by an additional 30 days in 6 individuals (Supplementary Materials).

### 2.4. Probiotic Compound and Placebo

Participants were randomly assigned to receive a 6-month course of oral supplementation with daily probiotics or placebo. The probiotic compound was composed as follows: *Streptococcus thermophilus* DSM24731®, *Lactobacillus plantarum* DSM24730®, *Bifidobacterium breve* DSM24732®, *Lactobacillus paracasei* DSM24733®, *Lactobacillus delbrueckii* subsp. *bulgaricus* DSM24734®, *Lactobacillus acidophilus* DSM 24735®, *Bifidobacterium longum* DSM24736®, *Bifidobacterium infantis* DSM24737®, (trade name: Vivomixx® in EU and Visbiome® in USA). Probiotics were administered at the concentration of 1800 billion bacteria/day (4 sachet/day, each sachet containing 450 billion bacteria cells in powder formulation). The placebo was composed of maltose and silicon dioxide. Sachet envelopes and content were indistinguishable between probiotics and placebo. Since probiotics needed refrigeration, storage at +4 °C was required for both compounds. All participants were told to ingest the study product at least 30 min before or 2 h after introducing food.

### 2.5. Statistical Analysis

Since literature data on the topic of the study are scarce and since this is a pilot study, the number of participants was arbitrarily set at 40 subjects. The interim analysis of 50% of the overall population to be enrolled in the trial and the final analysis with 100% of sample size enrolled were planned. Statistical analysis was conducted with Statistical Package for Social Science software, version 25 (SPSS Statistics, IBM, New York, NY, USA). Unless otherwise stated, data were presented as median and interquartile range (IQR, 25th–75th) for continuous variables, and as proportions, simple frequencies (n) and percentages for categorical variables. The Kolmogorov–Smirnov test and histograms were applied to assess the normal distribution of data. The presence of statistically significant differences between groups was assessed using Student t-test or Mann–Whitney test for normally and non-normally distributed variables, respectively. The Chi-square test (χ^2^) was used to test group differences of proportions.

Multivariate logistic regression models were used to adjust for potential confounding. Odds ratios (ORs) with 95% confidence intervals (95% CIs) were calculated for all associations. A two-sided *p*-value of less than 0.05 was considered statistically significant.

### 2.6. Ethical Aspects

The study was independently approved by the Committee of the Public Health and Infectious Diseases Department of “Sapienza” University of Rome and by the local Ethics Committee (N° of approval 457/17, 29 May 2017). Written informed consent was obtained from each participant prior to the enrollment in the trial. The study protocol was registered in the ClinicalTrials.gov register (Identifier number: NCT04099433).

Probiotic compound and placebo were donated by the probiotic manufacturing company without any obligation. The probiotic manufacturing company did not take part in data analysis nor did it obtain access to study data or results prior to their publication.

## 3. Results

Since the trial is still ongoing, here we report the interim analysis (as per protocol) of the results of the first 20 participants that concluded the protocol.

### 3.1. Baseline Characteristics of Participants

Among 20 subjects that have concluded the study protocol to date, 9 have been randomized to daily intake of oral probiotics and 11 to placebo. Baseline characteristics of the study population are reported in Table 1.

The concordance between anal biopsies and diagnosis of dysplasia was 100%. Total number of dysplasia observed at baseline was 17 in the experimental arm and 19 in the control arm. In each group, five subjects showed more than one lesion.

### 3.2. End of Study Results (T1)

All participants reported unprotected receptive anal intercourse during the study. 44.5% of participants in the experimental arm reported sexual contacts exclusively with the usual partner and 55.5% with multiple sexual partners. In the control arm, 36% of participants had receptive anal intercourses with the usual partner and 64% with multiple sexual partners.

At the end of the study, individuals in the experimental group showed significant improvements in the considered outcomes as compared to the control group, as shown below.

Nutritional and metabolic status of patients were also evaluated, as part of the routine follow-up for HIV+ individuals. No differences in blood pressure, body weight, BMI and lipid profile (cholesterol and triglycerides) were observed between groups at T1 (nor at baseline).

Main characteristics of the study population at T1 are reported in Table 2.

#### 3.2.1. Effects of Modulation of Gut Flora on Anal HPV Infection in HIV-Positive MSM

At T1, 67% of subjects in the experimental arm and 18% in the control group showed clearance of baseline anal HPV (*p* = 0.067).

In the experimental arm: three participants with hr-HPV showed a negative HPV DNA at T1 and three participants (two with hr-HPV and one with lr-HPV) showed clearance of baseline HPV infection but acquired a new genotype. In the control arm: two participants cleared baseline HPV infection but showed acquisition of a new genotype at T1.

#### 3.2.2. Effects of Modulation of Gut Flora on Anal Dysplasia in HIV-Positive MSM Anal Cytology

In the experimental arm: two participants with cytologic LSIL at baseline showed absence of abnormalities at T1. In the control arm: one participant with normal cytology at enrollment showed onset of LSIL at T1 and one participant with cytologic LSIL at baseline showed HSIL at T1 (HSIL was subsequently confirmed by histology).

#### 3.2.3. HRA and Histology Results

Among the population of the experimental arm: total number of dysplasia decreased from 17 to 12, the median number of lesions decreased from one with IQR 1–2 to one with IQR 0–1 and the number of participants with multiple dysplasia decreased from five to two. Persistence of dysplasia with unchanged HRA characteristics was observed in 55% of subjects, while persistence with worsening of HRA appearance was observed in 11% of participants.

Among the population of the control arm: total number of dysplasia increased from 19 to 30, the median number of lesions increased from two with IQR 1–3) to two with IQR 2–4 and the presence of multiple dysplasia increased from five to nine participants. Persistence of dysplasia with unmodified characteristics at HRA was observed in 54% of participants, while persistence with worsening of HRA appearance was observed in 73% of participants.

The regression of at least one dysplastic lesion was observed in 88% of subjects in the experimental arm versus 9% in the control arm. At the end of the study a normal HRA with absence of abnormal area was observed in 33% of the experimental group population and 0% of the control group.

**Figure 2:**
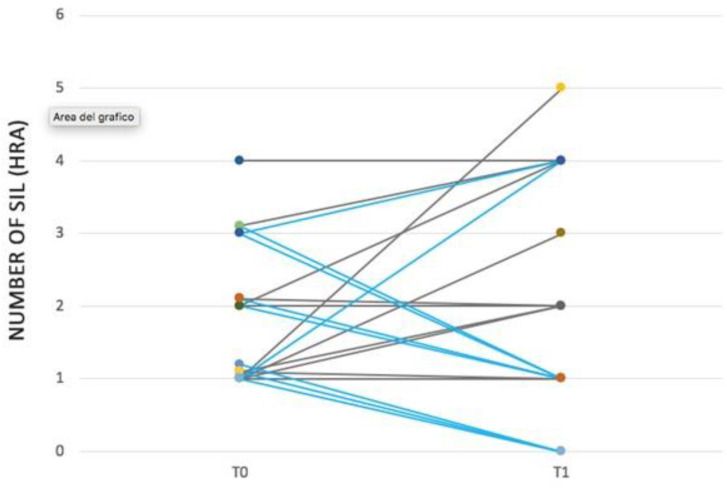
Number of SIL on HRA examination. Blue lines: experimental group. Grey lines: control group.

Onset of new dysplasia was observed in 22% of participants in the experimental arm (LSIL in both cases) and 73% of participants in the control arm, 37.5% of whom showed histology-proven HSIL. The number of SIL on HRA examination is shown in Figure 2.

At baseline, condyloma of the anal canal were observed in two participants in the experimental arm and one in the control arm. At T1, while regression was observed in all cases, onset of new condylomas was observed in one subject in the control arm.

Multivariate linear regression confirmed the observed results. In the study population, being assigned to probiotic intake was associated with an increased likelihood of HPV clearance (OR 8.9; *p* = 0.028) as well as an increased likelihood of regression of pre-existent dysplasia (OR 80; *p* = 0.001). Moreover, the intake of probiotic compound was protective against the onset of new lesions (OR 0.107; *p* = 0.025) and protective against worsening of persistent dysplasia (OR 0.047; *p* = 0.009).

#### 3.2.4. Safety and Tolerability of Oral Bacteriotherapy

No adverse events were reported during the course of the study. Participants in both groups completed a self-reported questionnaire on gastrointestinal symptoms at baseline and T1. No differences were observed in terms of tolerability between probiotic and placebo, with abdominal bloating during the first weeks reported by one participant in each group.

## 4. Discussion

The incidence of SCCA among HIV+ MSM can be as high as the incidence of prostate or colorectal cancer in the general population or even greater, depending on patients’ age [29]. Similarly, the prevalence of anal dysplasia among HIV+ MSM ranges between 50% and 90%, and it has been reported to be high also in young individuals [30].

Vaccination in HPV-infected subjects showed some effectiveness in the prevention of anal dysplasia recurrence, but its indication mainly refers to the prevention of HPV infection and anal precancers rather than its treatment. Early detection and ablation of anal cancer precursors has been adopted from many clinicians as the preferred strategy to prevent progression to SCCA [31]. The effectiveness of a similar approach has been proven to date for the prevention of HPV-related neoplasia of the female genital tract. Targeted HPV anti-viral drugs are not currently available and, even so, efficacy of such molecules against dysplasia could not be assumed without specific investigations.

The administration of lactic acid bacteria is of common use as aid in the treatment of a wide range of diseases, due to their local and systemic immunomodulatory effects. In women with bacterial vaginosis, the administration of lactic acid bacilli promotes a local HPV-unfavorable environment through the restoration of the integrity of epithelial lining, increase of mucus production, decrease of local pH and, most importantly, enhancement of immune response against HPV-infected cells. Moreover, in people living with HIV, probiotics demonstrated the ability to improve gut mucosa integrity, replenish immune cells of the mucosal compartment and compete with the overgrowth of detrimental saprophytic bacteria [32,33]. In this scenario, probiotics that contain lactic acid bacteria represent a potential and intriguing strategy to promote immune response against HPV and HPV-infected cells.

In synthesis in this study, 20 participants concluded the protocol procedures to date. Individuals in the experimental arm showed a higher rate of anal dysplasia regression (*p* = 0.002), lower rate of onset of new anal dysplasia (*p* = 0.023) and lower rates of worsening of persistent lesions (*p* = 0.004), with respect to participants randomized to placebo. Clearance of anal HPV infection was more frequently observed in the bacteriotherapy group (*p* = 0.067), however it did not reach statistical significance. No adverse effects or safety concerns were reported in the experimental group.

Major limitations of this interim analysis of the study were the small sample size of participants enrolled, short study interval and the weak p-values in the clearance of baseline anal HPV (*p* = 0.067) despite the trend observed (67% of subjects in the experimental arm and 18% in the control group).

Since this is an interim analysis, and considering its limits, we limit ourselves to reporting the preliminary results of the current study, without drawing conclusions. We refer the conclusions relating to the possible effectiveness of the intervention with this specific probiotic formulation to the analysis of the definitive data.

## 5. Conclusions

The findings of the interim analysis are in line with the evidence coming from similar studies that have been undertaken in female patients with HPV-related cervical lesions [15,25].

Although seemingly encouraging and worthy of disclosure, these results are nevertheless too preliminary and therefore potentially biased to draw ultimate conclusions.

## Figures and Tables

**Table 1:** Baseline characteristics (T0) of the study population. Data are reported as total number (%) ore median (IQR).

	Experimental Group	Control Group	*p*-Value
Participants	9	11	
Age	44 (35–49.5)	55 (50–58)	0.010
Diabetes	0	1 (9%)	0.341
Cigarette smokers	3 (33%)	6 (55%)	0.367
Anal receptive intercourse	9 (100%)	11 (100%)	1
Lifetime sexual partners	100 (100–150)	50 (40–250)	0.341
HPV vaccination	0	0	1.000
Years from HIV diagnosis	12 (10–14)	18 (10.5–25.5)	0.081
T CD4 nadir (cells/μL)	460 (350–532)	262 (113–406)	0.387
T CD4 baseline (cells/μL)	877 (700–936)	700 (540–897)	0.606
Treatment with NRTI	8 (89%)	9 (82%)	0.673
Treatment with INSTI	4 (44%)	8 (73%)	0.227
Treatment with NNRTI	5 (56%)	3 (27%)	0.227
Treatment with PI	1 (11%)	2 (18%)	0.673
High risk HPV	6 (67%)	5 (45%)	0.367
Anal condyloma	2 (22%)	1 (9%)	0.656
Number of SIL (histology)	1 (1–2)	2 (1–3)	0.714
Participants with HSIL (histology)	1 (11%)	0	0.317

NRTI: nucleoside reverse transcriptase inhibitors; INSTI: integrase strand transfer inhibitors; NNRTI: non nucleoside reverse transcriptase inhibitors; PI: protease inhibitors.

**Table 2:** Characteristic of the two groups at the end of the study (T1). Data are reported as total number (%) ore median (IQR).

	Experimental Group	Control Group	*p*-Value
HPV clearance	6 (67%)	2 (18%)	0.067
High risk HPV T1	3 (33%)	6 (55%)	0.367
Number of SIL (histology)	1 (0–1)	2 (2–4)	0.053
Participants with normal HRA	3 (33%)	0	0.230
Participants with regression of at least 1 SIL	8 (88%)	1 (9%)	0.002
Participants with persistent and worsened SIL	1 (11%)	7 (78%)	0.004
Participants with onset of new SIL	2 (22%)	8 (73%)	0.023
Participants with HSIL (histology)	0	3 (27%)	0.083

## Data Availability

Data will be available on request to the first author (eugenionelson.cavallari@uniroma1.it).

## Supplementary Materials

The following are available online at https://www.mdpi.com/article/10.3390/biomedicines9111738/s1, Figure S1: Regression of high-grade lesion (HSIL) in a participant in the experimental arm. A: acetic acid appearance of the lesion at T0. B: Lugol-iodine appearance of the lesion at T0. C: acetic acid appearance of the area at T1. D: Lugol-iodine appearance of the area at T1, Figure S2: Progression of a dysplastic lesion in a participant in the observational arm. A: acetic acid appearance of the lesion at T0 (LSIL). B: Lugol-iodine appearance of the lesion at T0 (LSIL). C: acetic acid appearance of the area at T1 (HSIL). D: Lugol-iodine appearance of the area at T1 (HSIL).

## Abbreviations

Squamous cell carcinoma of the anus	SCCA
Human Papilloma Virus	HPV
low grade squamous intraepithelial lesions	LSIL
high grade squamous intraepithelial lesions	HSIL
men having sex with men	MSM
randomized controlled trial	RCT
highly active anti-retroviral therapy	HAART
high resolution anoscopy	HRA
nucleoside reverse transcriptase inhibitors	NRTI
integrase strand transfer inhibitors	INSTI
non nucleoside reverse transcriptase inhibitors	NNRTI
protease inhibitors	PI

## References

[1] Kjaer S.K., van den Brule A.J., Bock J.E., Poll P.A., Engholm G., Sherman M.E., Walboomers J.M., Meijer C.J. (1996). Human papillomavirus-the most significant risk determinant of cervical intraepithelial neoplasia. Int. J. Cancer.

[2] Schiffman M., Bauer H.M., Hoover R., Glass A.G., Cadell D.M., Rush B.B., Scott D.R., Sherman M., Kurman R., Wacholder S.C. (1993). Epidemiologic evidence showing the HPV infection causes most cervical intraepithelial neoplasia. J. Natl. Cancer Inst..

[3] Moscicki A.B., Ma Y., Farhat S., Jay J., Hanson E., Benningfield S., Jonte J., Godwin-Medina C., Wilson R., Shiboski S. (2014). Natural history of anal Human Papillomavirus infection in heterosexual women and risks associated with persistence. Clin. Infect. Dis..

[4] Ellerbrock T.V., Chiasson M.A., Bush T.J., Sun X.W., Sawo D., Brudney K., Wright T.C. (2000). Incidence of cervical squamous intraepithelial lesions in HIV-infected women. JAMA.

[5] Mandelblatt J.S., Fahs M., Garibaldi K., Senie R.T., Peterson H.B. (1992). Association between HIV infection and cervical neoplasia: Implications for clinical care of women at risk for both conditions. AIDS.

[6] Palefsky J.M., Minkoff H., Kalish L.A., Levine A., Sacks H.S., Garcia P., Young M., Melnick S., Miotti P., Burk R. (1999). Cervicovaginal human papillomavirus infection in human immunodeficiency virus-1 (HIV)-positive and high-risk HIV-negative women. J. Natl. Cancer Inst..

[7] Wang C.J., Palefsky J.M. (2019). HPV-Associated Anal Cancer in the HIV/AIDS Patient. Cancer Treat Res..

[8] Wu P.F., Hang J.F., Strong C., Chen S.J., Lin L.Y., Chen S.S., Lai C.R., Ku S.W., Lee M.H. (2020). Anal human papillomavirus and its associations with abnormal anal cytology among men who have sex with men. Sci Rep..

[9] Hanson L., Vande V.L., Jermé M., Abad C.L., Safdar N. (2016). Probiotics for Treatment and Prevention of Urogenital Infections in Women: A Systematic Review. J. Midwifery Womens Health.

[10] MacPhee R.A., Hummelen R., Bisanz J.E., Miller W.L., Reid G. (2010). Probiotic strategies for the treatment and prevention of bacterial vaginosis. Expert Opin. Pharmacother..

[11] Reid G., Dols J., Miller W. (2009). Targeting the vaginal microbiota with probiotics as a means to counteract infections. Curr. Opin. Clin. Nutr. Metab. Care.

[12] Spurbeck R., Arvidson C.G. (2011). Lactobacilli at the front line of defence against vaginally acquired infections. Future Microbiol..

[13] Bolton M., van der Straten A., Cohen C.R. (2008). Probiotics: Potential to prevent HIV and sexually transmitted infections in women. Sex Transm. Dis..

[14] Verhoeven V., Renard N., Makar A., Van Royen P., Bogers J.P., Lardon F., Baay M. (2013). Probiotics enhance the clearance of human papillomavirus-related cervical lesions: A prospective controlled pilot study. Eur. J. Cancer Prev..

[15] Palma E., Recine N., Domenici L., Giorgini M., Pierangeli A., Panici P.B. (2018). Long-term Lactobacillus rhamnosus BMX 54 application to restore a balanced vaginal ecosystem: A promising solution against HPV-infection. BMC Infect Dis..

[16] Xiong Y., Cui L., Bian C., Zhao X., Wang X. (2020). Clearance of human papillomavirus infection in patients with cervical intraepithelial neoplasia: A systemic review and meta-analysis. Medicine.

[17] Wang K.D., Xu D.J., Wang B.Y., Yan D.H., Lv Z., Su J.R. (2018). Inhibitory Effect of Vaginal Lactobacillus Supernatants on Cervical Cancer Cells. Probiotics Antimicrob. Proteins.

[18] Abdolalipour E., Mahooti M., Salehzadeh A., Torabi A., Mohebbi S.R., Gorji A., Ghaemi A. (2020). Evaluation of the antitumor immune responses of probiotic Bifidobacterium bifidum in human papillomavirus-induced tumor model. Microb. Pathog..

[19] Abdolalipour E., Mahooti M., Gorji A., Ghaemi A. (2020). Synergistic Therapeutic Effects of Probiotic *Lactobacillus casei TD-2* Consumption on GM-CSF-Induced Immune Responses in a Murine Model of Cervical Cancer. Nutr. Cancer..

[20] Zevin A.S., McKinnon L., Burgener A. (2016). Microbial translocation and microbiome dysbiosis in HIV-associated immune activation. Curr. Opin. HIV AIDS.

[21] Vujkovic-Cvijin I., Dunham R.M., Iwai S. (2013). Dysbiosis of the gut microbiota is associated with HIV disease progression and tryptophan catabolism. Sci. Transl. Med..

[22] Dillon S.M., Lee E.J., Kotter C.V. (2014). An altered intestinal mucosal microbiome in HIV-1 infection is associated with mucosal and systemic immune activation and endotoxemia. Mucosal Immunol..

[23] Brenchley J.M., Price D.A., Schacker T.W. (2006). Microbial translocation is a cause of systemic immune activation in chronic HIV infection. Nat. Med..

[24] Ceccarelli G., Cavallari E.N., Savinelli S., Bianchi L., Pierangeli A., Vullo F., Ciardi A., D'ettorre G. (2018). Clearance of human papillomavirus related anal condylomas after oral and endorectal multistrain probiotic supplementation in an HIV positive male: A case report. Medicine.

[25] Verteramo R., Pierangeli A., Mancini E., Calzolari E., Bucci M., Osborn J., Nicosia R., Chiarini F., Antonelli G., Degener A.M. (2009). Human papillomaviruses and genital co-infections in gynecological outpatients. BMC Infect. Dis..

[26] Taylor S., Bunge E., Bakker M., Castellsagué X. (2016). The incidence, clearance and persistence of non-cervical human papillomavirus infections: A systematic review of the literature. BMC Infect Dis..

[27] Darragh T.M., Colgan T.J., Cox J.T., Heller D.S., Henry M.R., Luff R.D., McCalmont T., Nayar R., Palefsky J.M., Stoler M.H. (2012). Members of LAST Project Work Groups. The Lower Anogenital Squamous Terminology Standardization Project for HPV-Associated Lesions: Background and consensus recommendations from the College of American Pathologists and the American Society for Colposcopy and Cervical Pathology. Arch. Pathol. Lab. Med..

[28] Hillman R.J., Cuming T., Darragh T., Nathan M., Berry-Lawthorn M., Goldstone S., Law C., Palefsky J., Barroso L.F., Stier E.A. (2016). 2016 IANS International Guidelines for Practice Standards in the Detection of Anal Cancer Precursors. J. Low Genit. Tract. Dis..

[29] Clifford G.M., Georges D., Shiels M.S., Engels E.A., Albuquerque A., Poynten I.M., de Pokomandy A., Stier E.A. (2021). A meta-analysis of anal cancer incidence by risk group: Toward a unified anal cancer risk scale. Int. J. Cancer..

[30] Palefsky J.M., Lensing S.Y., Belzer M., Lee J., Gaur A.H., Mayer K., Futterman D., Stier A., Paul M.E., Chiao E.Y. (2021). High Prevalence of Anal High-Grade Squamous Intraepithelial Lesions, and Prevention Through Human Papillomavirus Vaccination, in Young Men Who Have Sex With Men Living With Human Immunodeficiency Virus. Clin. Infect. Dis..

[31] Wieland U., Kreuter A. (2013). One step towards standardized management of anal dysplasia. Lancet Oncol..

[32] Liu J., Williams B., Frank D., Dillon S.M., Wilson C.C., Landay A.L. (2017). Inside Out: HIV, the Gut Microbiome, and the Mucosal Immune System. J. Immunol..

[33] Ceccarelli G., Statzu M., Santinelli L., Pinacchio C., Bitossi C., Cavallari E.N., Vullo V., Scagnolari C., d’Ettorre G. (2019). Challenges in the management of HIV infection: Update on the role of probiotic supplementation as a possible complementary therapeutic strategy for cART treated people living with HIV/AIDS. Expert Opin. Biol. Ther..

