# Supplementary material for: Clinical Effects of Oral Bacteriotherapy on Anal HPV Infection and Related Dysplasia in HIV-Positive MSM: Results from the “HPVinHIV” Trial
Source: Biomedicines. 2021 Nov 22;9(11):1738. doi: 10.3390/biomedicines9111738 (PMC8615833; doi:10.3390/biomedicines9111738)

Supplementary Materials:

Figure S1: Regression of high-grade lesion (HSIL) in a participant in the experimental arm. **A**: acetic acid appearance of the lesion at T0. **B**: Lugol-iodine appearance of the lesion at T0. **C**: acetic acid appearance of the area at T1. **D**: Lugol-iodine appearance of the area at T1.

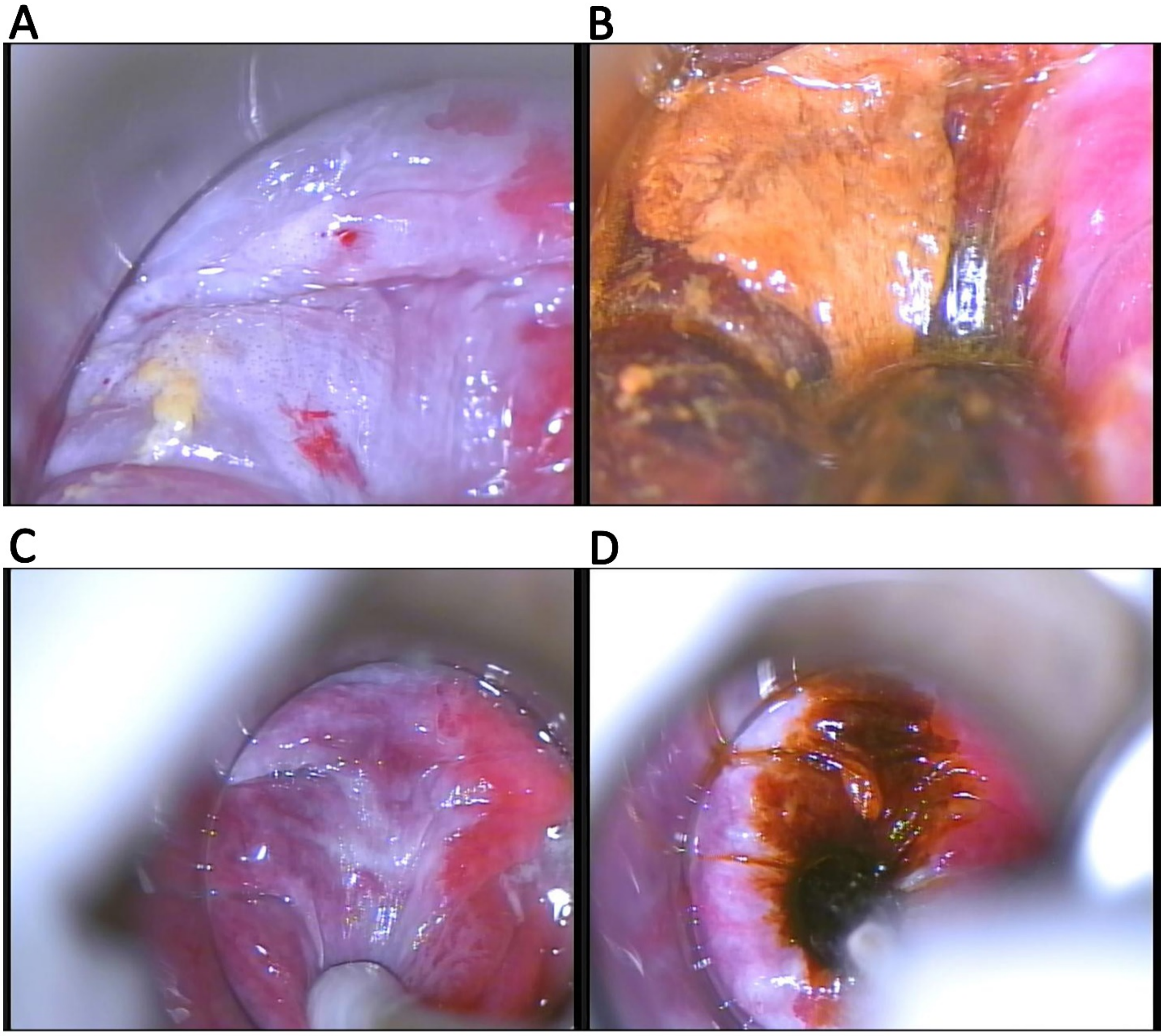

Figure S2: Progression of a dysplastic lesion in a participant in the observational arm. **A**: acetic acid appearance of the lesion at T0 (LSIL). **B**: Lugol-iodine appearance of the lesion at T0 (LSIL). **C**: acetic acid appearance of the area at T1 (HSIL). **D**: Lugol-iodine appearance of the area at T1 (HSIL).

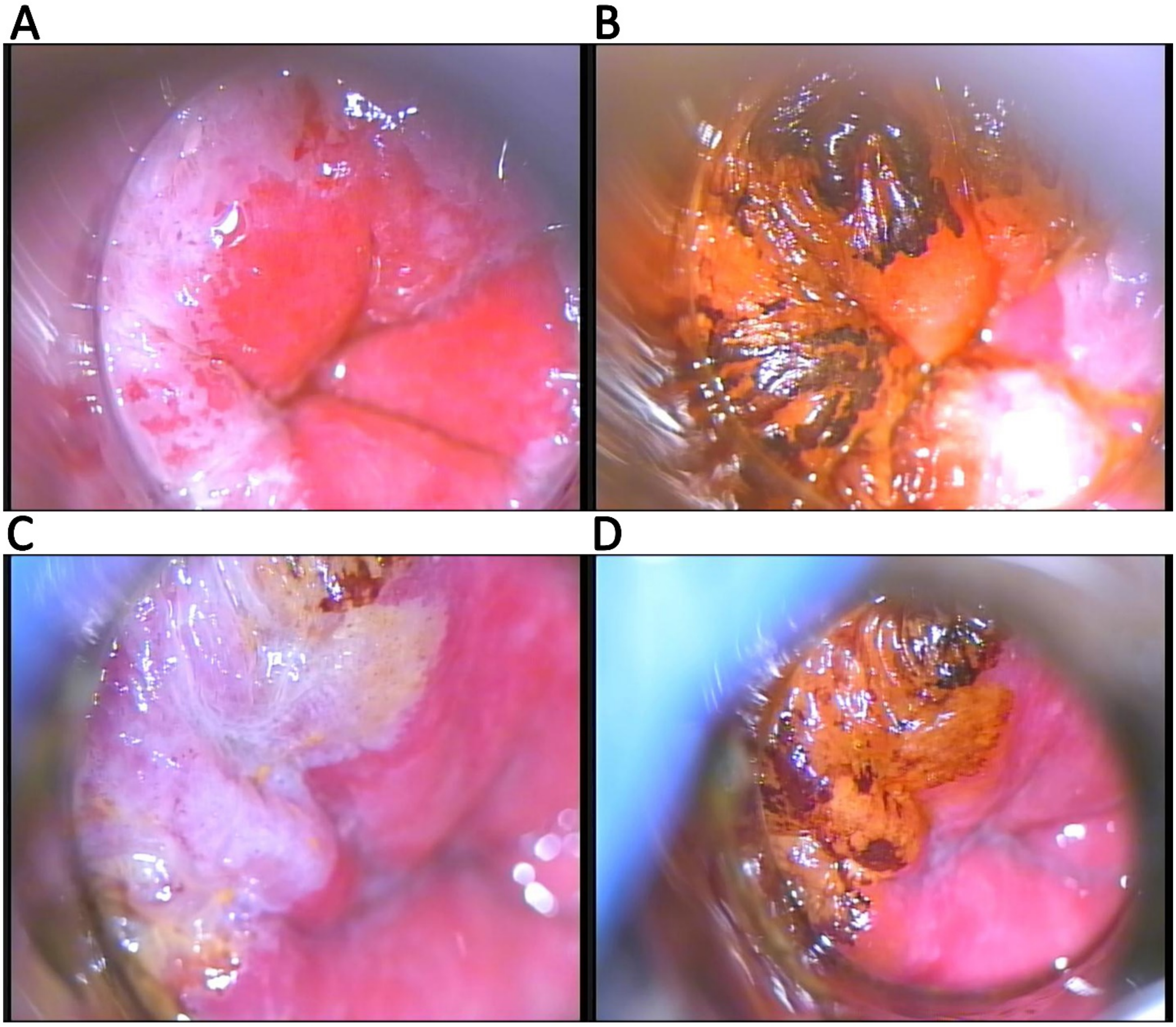

Supplement: Supplementary file 1 [file biomedicines-09-01738-s001.zip › biomedicines-1431351-supplementary.pdf]
